# Luminal and mucosa-associated caecal microbiota of chickens after experimental *Campylobacter jejuni* infection in the absence of *Campylobacter*-specific phages of group II and III

**DOI:** 10.1099/mgen.0.000874

**Published:** 2022-10-03

**Authors:** Julia Hankel, Sophie Kittler, Bussarakam Chuppava, Eric Galvez, Till Strowig, André Becker, Maren von Köckritz-Blickwede, Madeleine Plötz, Christian Visscher

**Affiliations:** ^1^​ Institute for Animal Nutrition, University of Veterinary Medicine Hannover, Foundation, Bischofsholer Damm 15, D 30173 Hannover, Germany; ^2^​ Institute for Food Quality and Food Safety, University of Veterinary Medicine Hannover, Foundation, Bischofsholer Damm 15, D 30173 Hannover, Germany; ^3^​ Helmholtz Center for Infection Research, Inhoffenstraße 7, D 38124 Braunschweig, Germany; ^4^​ Hannover Medical School, Carl-Neuberg-Straße 1, D 30625 Hannover, Germany; ^5^​ Research Center for Emerging Infections and Zoonoses, University of Veterinary Medicine Hannover, Foundation, Bünteweg 17, D 30559 Hannover, Germany

**Keywords:** bacteriophages, Campylobacter, gut microbiota, mucus, poultry, 16S rRNA genes

## Abstract

Campylobacteriosis is still the most commonly reported zoonosis in the European Union causing gastrointestinal disease in humans. One of the most common sources for these food-borne infections is broiler meat. Interactions between *Campylobacter* (*C*.) *jejuni* and the intestinal microbiota might influence *Campylobacter* colonization in chickens. The aim of the present study was to gain further knowledge about exclusive interactions of the host microbiota with *C. jejuni* in *Campylobacter *-specific phage-free chickens under standardized conditions and special biosafety precautions.

Therefore, 12 artificially infected (* C. jejuni* inoculum with a challenge dose of 7.64 log_10_ c.f.u.) and 12 control chickens of the breed Ross 308 were kept under special biosafety measures in an animal facility. At day 42 of life, microbiota studies were performed on samples of caecal digesta and mucus. No *Campylobacter *-specific phages were detected by real-time PCR analysis of caecal digesta of control or artificially infected chickens. Amplification of the 16S rRNA gene was performed within the hypervariable region V4 and subsequently sequenced with Illumina MiSeq platform. R (version 4.0.2) was used to compare the microbiota between *C. jejuni *-negative and *C. jejuni *-positive chickens. The factor chickens’ infection status contributed significantly to the differences in microbial composition of mucosal samples, explaining 10.6 % of the microbiota variation (*P*=0.007) and in digesta samples, explaining 9.69 % of the microbiota variation (*P*=0.015). The strongest difference between *C. jejuni *-non-infected and *C. jejuni *-infected birds was observed for the family *Peptococcaceae* whose presence in *C. jejuni *-infected birds could not be demonstrated. Further, several genera of the family *Ruminococcaceae* appeared to be depressed in its abundance due to *Campylobacter* infection. A negative correlation was found between *Christensenellaceae* R-7 group and *Campylobacter* in *C. jejuni *-colonised chickens, both genera potentially competing for substrate. This makes *Christensenellaceae* R-7 group highly interesting for further studies that aim to find control options for *Campylobacter* infections and assess the relevance of this finding for chicken health and *Campylobacter* colonization.

## Data Summary

The following supplementary data are available. Table S1: Relative abundance (%) of bacterial phyla in luminal or mucosa-associated microbiota depending on *C. jejuni* status. Table S2: Comparison of bacterial relative abundance in samples of caecal content and mucus (at taxonomic level, family) between chickens of different *C. jejuni* status. Table S3: OTUs with significant (padj <0.05) different abundance between chickens of different *C. jejuni* status in samples of caecal content. Table S4: OTUs with significant (padj <0.05) different abundance between chickens of different *C. jejuni* status in samples of caecal mucus. Table S5: Real-time PCR results. Table S6: Sample overview.

The authors confirm all supporting data and protocols have been provided within the article or through supplementary data files.

**Impact Statement**

Preliminary studies have shown interactions between intestinal microbiota and *Campylobacter* spp. influencing host susceptibility against this pathogen. As intestinal microbiota seems to impact *Campylobacter* (*C*.) *jejuni* colonization, load and clearance, the long-term goal is to identify bacterial species or specific microbiota compositions that might promote colonization resistance. Any reduction in *Campylobacter* load in chicken intestines is a further step towards increasing food safety because campylobacteriosis is the most commonly reported zoonosis in the European Union causing gastrointestinal disease. However, further knowledge is needed concerning interactions of the host microbiota and *C. jejuni *. When examining these relationships, it is important to ensure the absence of naturally occurring *Campylobacter *-specific bacteriophages, as they are present in about one third of *C. jejuni* positive conventional barn-reared broiler chickens and their presence is associated with a significant reduction in *C. jejuni* counts. For this reason, the present study was conducted under special biosafety measures. This study also explores possible interactions within the caecal luminal and mucosa-associated microbiota under standardized conditions and biosafety precautions, which is why the study could reveal important knowledge about exclusive interactions of the host microbiota and *C. jejuni *. The results of this study contribute to the goal of finding further control options for *Campylobacter* infections in chickens.

## Introduction

In 2019, campylobacteriosis was the most commonly reported zoonosis in the European Union causing gastrointestinal disease in humans, while one of the most common sources for the food-borne infections was broiler meat [1]. For this reason, a process hygiene criterion was established for *Campylobacter* in 2017 [2], putting pressure on broiler meat producers to reduce the *Campylobacter* load. Chilled broiler carcases should comply with a limit of 1000 c.f.u. g^−1^. However, currently no efficient control measures are available. Thus, control options for *Campylobacter* in broilers, focusing on primary production are still of major interest [3]. Since *Campylobacter (C.) jejuni* colonization in chickens is highly prevalent but asymptomatic, it appears to act as a commensal in this host (reviewed in Newell [4]). Various studies on animals and humans have shown that the composition of the intestinal microbiota plays a significant role in host susceptibility to *Campylobacter* species [5–8]. In humans, for example, a less diverse faecal microbiota was associated with a higher susceptibility to *Campylobacter* infections [8]. Even in chickens, studies revealed an influence of the microbiota on the outcome of a *Campylobacter* infection [5–7]. Compared to their hatchmates, higher *C. jejuni* counts and histopathological gut lesions were found in chickens that were raised under germ-free conditions or treated with an antibiotic cocktail [7]. *C. jejuni* invades the caecum of antibiotic-treated birds with limited gut microbiota and induces clinical signs and lesions, leading the authors to suggest that *C. jejuni* is a primary pathogen for chickens as well [6].

As intestinal microbiota seem to have an influence on *C. jejuni* colonization, load and clearance, the long-term goal is to identify bacterial species or specific microbiota compositions that could induce the colonization resistance. However, these efforts still require further knowledge concerning interactions of *C. jejuni* and the host microbiota.

Characteristics of *C. jejuni* suggest an adaptation to the environment of the mucus, which might also influence *C. jejuni* pathogenicity [9]. In addition, distinct community structures are observed in the luminal and mucosal samples, which necessitate studying the variations between the bacterial communities of the lumen and mucosa, thus improving the understanding of host–microbe interactions [10].

Along with bacteria, viruses are present in the chicken’s intestines that can influence *Campylobacter* [11]. It could be shown that naturally occurring *Campylobacter *-specific lytic bacteriophages are present in about one third of *C. jejuni* positive caecal samples of conventional barn-reared broiler chickens, and *C. jejuni* counts in the presence of bacteriophages were associated with a significant reduction [11].

The aim of the present study was to investigate the luminal and mucosa-associated caecal microbiota of experimentally *C. jejuni *-infected chickens under the absence of *Campylobacter *-specific phages. Results from this study may help to better understand the interactions of the host microbiota and *C. jejuni* in chickens, with the aim of finding further control options for primary production in the long term.

## Methods

A total of 24 newly hatched chickens (day 0) of both sexes (Ross 308) were obtained from a commercial hatchery (BWE-Brüterei Weser Ems, PHW Gruppe/Lohmann and Co. AG, Visbek, Germany). They were kept in two separate rooms in a biosafety level (BSL) 2 animal facility of the Research Center for Emerging Infections and Zoonoses (RIZ) at the University of Veterinary Medicine Hannover, Foundation, Hanover, Germany. The RIZ animal facility including BSL-2 stables harbours a special technology to reduce the pressure in all labs/stables. Directional air flow is established from clean areas into contaminated areas. If multiple containment zones exist within an area, sequentially more negative pressure differentials are established so that the more contaminated spaces are maintained at a negative pressure with respect to less contaminated areas. Access to the area was controlled and restricted to designated employees. Effective control of vectors was accomplished via a threefold barrier system. All items brought into the rooms were subjected to germ reduction measures. Each room had its own equipment. In both rooms, an identical box was installed, which was divided by a continuous wall and the 12 birds were kept first exclusively on one side of the box. Each half of the box was littered with wood shavings (1 kg/m^2^). Stocking density amounted to a maximum of 30 kg per square metre. The birds were maintained under a 16-hour light, 8-hour dark lighting schedule during the entire experiment.

The animals were fed in three phases with conventional complete diets. The starter diet was offered in the first week of life, the first grower diet in the second week of life and the second grower diet from day 14 onwards. The diets were analysed by standard procedures in accordance with the official methods of the VDLUFA [12] as described in Visscher, Klingenberg [13]. The second grower diet was mainly composed of wheat, extracted soybean meal, corn, extracted rapeseed meal, peas, soybean oil, and palm oil and contained 19.7 % crude protein and 12.5 MJ AME per kg diet.

**Fig. 1.**
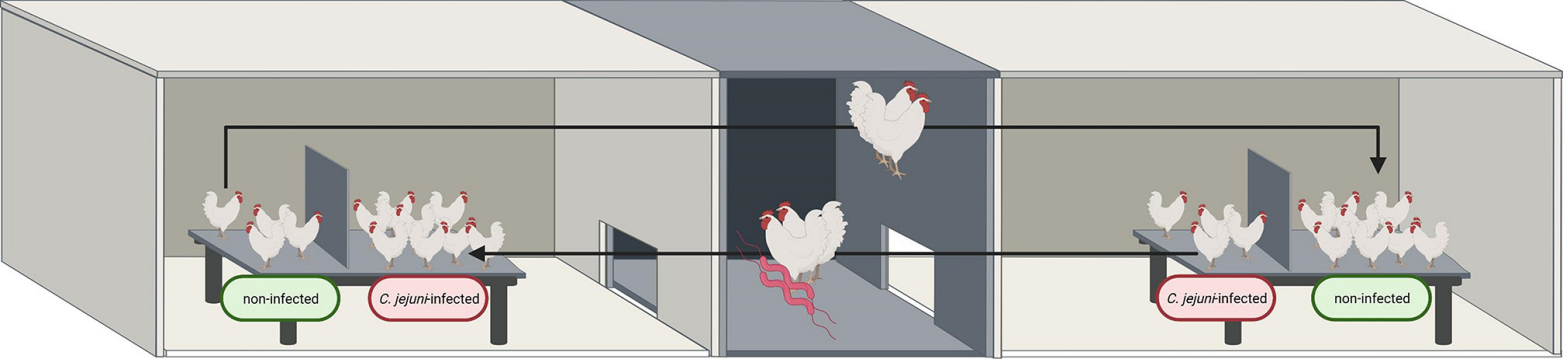
Chickens kept in two rooms under special biosafety measures in the animal facility. Every 3 days, two animals from each box changed rooms. The continuous wall prevented direct contact between the artificially *C. jejuni *-infected and non-infected birds kept in one box. The figure was created with biorender.com.

At day 17 of life, each bird in the first room (12 birds) was administered orally with one millilitre of a *C. jejuni* inoculum (challenge dose of 7.64 log_10_ c.f.u./1 ml of challenge inoculum). The *C. jejuni* inoculum was a mixture of the *C. jejuni* strain C356 (DSM 24306, Leibniz Institute DSMZ – German Collection of Microorganisms and Cell Cultures, Braunschweig, Germany) and a strain that was isolated from sheddings of chickens participating in a previous microbiota study by Hankel *et al*. [5]. Prior to the described experimental challenge, *Campylobacter* exclusion diagnosis had been performed via qualitative bacteriological examination. In order to ensure identical environmental and at the same time to guarantee controlled infection conditions for each animal (non-infected and artificially infected birds), two animals from each box changed rooms every 3 days (see Fig. 1). The continuous wall prevented direct contact between the non-infected and artificially infected birds when kept in one box during the whole experiment. This effort guaranteed that each animal was kept once in each room and box, therefore exposed to identical environmental conditions.

Three weeks after the experimental challenge with *C. jejuni *, all chickens were weighed and dissected (day 42). Anaesthesia was performed by head stroke. After bleeding, caecal contents were removed under sterile conditions and placed in reaction vessels. The caecal contents were immediately frozen and stored at −80 °C for further microbiota analyses and examination of the presence or absence of *Campylobacter *-specific bacteriophages. In addition, mucus samples from the caecum were collected in accordance with the protocol described below.

### Bacteriological analyses

Qualitative bacteriological examination of *C. jejuni* performed in caecal contents of all birds was based on the DIN EN ISO 10272–1 : 2006, taken from the official collection of analysis methods in accordance with § 64 LFBG. Quantitative bacteriological examination was performed as described in Hankel *et al.* [5].

### *Campylobacter *-specific bacteriophages

Detection of *Campylobacter *-specific group II and III bacteriophages in caecal content was carried out as described by Jäckel *et al*. [14] with some modifications. In brief, primers and probes were received from biomers.net (biomers.net GmbH, Ulm, Germany), using primer and probe sequences described by Jäckel *et al*. [14]. For real-time PCR, a 1 g sample of each caecal content was thoroughly mixed with 9 ml of 0.9 % sodium chloride and centrifuged at 13.000 ***g*** for 10 min at 4 °C to remove debris. Subsequently, the supernatant was heated at 95 °C for 20 min. For CPGIII- and CPGII/GIII-detection, multiplex real-time PCR was used: 1 µl of each sample suspension was mixed with 5 µl QuantiNova Multiplex PCR Master Mix (Quiagen GmbH, Hilden, Germany), each 0.8 µl of CPGIII and CPGII/GIII forward and reverse primers, 0.25 µl CPGIII and CPGII/GIII probe and 10.30 µl water. Amplification was done as described for the QuantiNova Multiplex PCR Kit. For CPGII-detection, one microlitre of each sample suspension was mixed with 5 µl QuanitNova Multiplex PCR Master Mix, each 0.8 µl of CPGII forward and reverse primers, 0.25 µl CPGII probe and 12.15 µl water. The amplification conditions were as follows: initial denaturation at 95 °C for 2 min followed by 40 cycles of 95 °C for 5 s, and 55 °C for 30 s. Reactions were performed using the LightCycler 480 instrument II (Roche Diagnostics GmbH, Mannheim, Germany). Analysis of ct values and fluorescence emission intensity curves was performed using LightCycler 480 software Release 1.5OSP3 IDEAS 2.0. A ct value cut-off of ≤37 as validated by Jäckel *et al*. [14] was used. Samples with ct values above 37 or no rising fluorescence emission intensity curves were considered to contain no *Campylobacter *-specific group II or III phages (Table S5).

### Preparation of mucosal samples

Cell-wall-associated bacteria were obtained based on the described procedure by Gong *et al*. [15]. Both caeca were opened longitudinally and immediately washed in saline three times, then washed twice in saline containing 0.1 % Tween 80 under vigorous hand shaking for 30 s per wash. In contrast to the method described, only the last washing solution was centrifuged (27000 ***g***, 20 min) at 4 °C to pellet the bacterial cells that were released from the caecal wall. This fraction of bacterial cells was referred to as mucosa-associated bacteria in the present investigation and immediately frozen and stored at −80 °C for further microbiota analyses.

## 16S rRNA gene sequencing

### DNA extraction

A total of 48 samples of caecal contents and mucus taken from 24 birds were immediately frozen and stored until simultaneous analysis at a temperature of −80 °C. A phenol-chloroform based protocol was used to isolate total DNA from samples. Therefore, the obtained samples were centrifuged (3500 r.p.m.) and suspended with 500 µl of extraction buffer (200 mM Tris, 20 mM EDTA, 200 mM NaCl, pH 8.0), 200 µl of 20 % SDS (sodium dodecyl sulphate), 500 µl of phenol:chloroform:isoamyl alcohol (24 : 24 : 1) and 100 µl of zirconia/silica beads (0.1 mm diameter). The samples were homogenized with a bead beater (BioSpec) for 2 min, DNA was precipitated with absolute isopropanol and washed with 70 % vol. ethanol. DNA extracts were suspended in TE Buffer with 100 µg ml^−1^ RNAse I and additionally column purified. Finally, total DNA was quantified and diluted to 25 ng µl^−1^.

### Sequencing and data processing

16S rRNA gene analysis was performed as described in Hankel *et al*. [5]. A purification step (Kit: BS 365, Bio Basic, Markham, Ontario, Canada) was performed before the hypervariable region V4 of the 16S rRNA gene was amplified in accordance with previously described protocols using the primer pair F515 (5′-GTGCCAGCMGCCGCGGTAA-3′) and R806 (3′-TAATCTWTGGGVHCATCAGG-5′) [16]. Amplicons were sequenced on the Illumina MiSeq platform (PE250) and the Usearch8.1 software package (http://www.drive5.com/usearch/) was used to assemble, quality control and cluster the obtained reads. The reads were merged and chimeric sequences were identified and removed. Quality filtering was set up with fastq_filter (-fastq_maxee 1) according to a minimum read length of 200 bp before reads were clustered into 97 % ID operational taxonomic units (OTUs). OTU clusters and representative sequences were determined with the UPARSE algorithm [17]. Taxonomy assignment was carried out with the help of Silva database v128 [18] and the Naïve Bayesian Classifier from the Ribosomal Database Project (RDP) [19]. The bootstrap confidence cut-off was set at 70 %.

Samples with fewer than 999 total reads were removed. Therefore, 47 out of 48 samples were included in statistical analyses of microbiota. The dataset contained 1 529 785 reads (mean number of reads: 32548; range: 17 644–40 847) mapped to 191 OTUs. The rarefaction curves were plotted using with the R-package ‘vegan’ (version 2.5.6, Figure S1).

### Statistical analyses

Statistical analyses of microbiota were performed using R (version 4.0.2, www.r-project.org) with the R-package ‘phyloseq’ (version 1.32.0 [20]). Ordination was performed using Bray–Curtis dissimilarity-based principal coordinate analysis (PCoA) also provided in the R-package ‘phyloseq’. Factors contributing to the differences in microbial composition of the samples were identified with permutational multivariate analysis of variance (PERMANOVA) on Bray–Curtis distances via the adonis function of the ‘vegan’ package (version 2.5.6 [21]), whereby to evaluate the contribution of the factor *C. jejuni* infection, OTU_90 (genus *Campylobacter *) was pruned from the dataset. Sample diversity was measured with the species richness estimators Observed Species, Chao 1 and Shannon index. Data were checked for normality by analysing the model residuals with the Shapiro–Wilk normality test before pairwise comparisons were conducted, all implemented in the package ‘rstatix’ (version 0.6.0 [22]). To identify bacterial phyla with significantly different abundance, multiple testing also included in the R-package ‘phyloseq’ was used on normalized counts. *P*-values from this test were adjusted by the Benjamini and Hochberg (BH) method to control for the false discovery rate (FDR) of 5 %. To find OTUs with significantly different abundance between birds, abundance counts were compared using the R-package ‘DESeq2’ (version 1.22.2), which uses tests based on the negative binomial distribution [23]. OTUs were filtered using false discovery rate (FDR) cut-off 0.05. Results were visualized with the help of ggplot2 (version 3.3.5 [24]). Finally, relative abundance of bacterial genera within luminal and mucosa-associated microbiota in *C. jejuni *-positive chickens that had a relative abundance >1 % in at least one sample were tested for association with the R-package ‘corrplot’ (version 0.84 [25]) using Spearman’s rank correlation.

## Results

### Performance data

The experiments ran without complications. The final body weight at day 42 amounted on average to 3 015 g±313 (* C. jejuni *-negative birds) and 3 140 g±361 (* C. jejuni *-positive birds) exceeding performance objectives of Aviagen at day 42 [26].

### Bacteriological examination and detection of *Campylobacter *-specific bacteriophages

Qualitative bacteriological examination revealed all caecal samples of experimentally *C. jejuni *-infected birds as being *C. jejuni* positive (5.31±0.69 log_10_ c.f.u. g^−1^), while test results of the non-infected birds were *C. jejuni* negative.

The presence of *Campylobacter *-specific phages of group II and III could be excluded.

### Luminal and mucosa-associated caecal microbiota

**Fig. 2.**
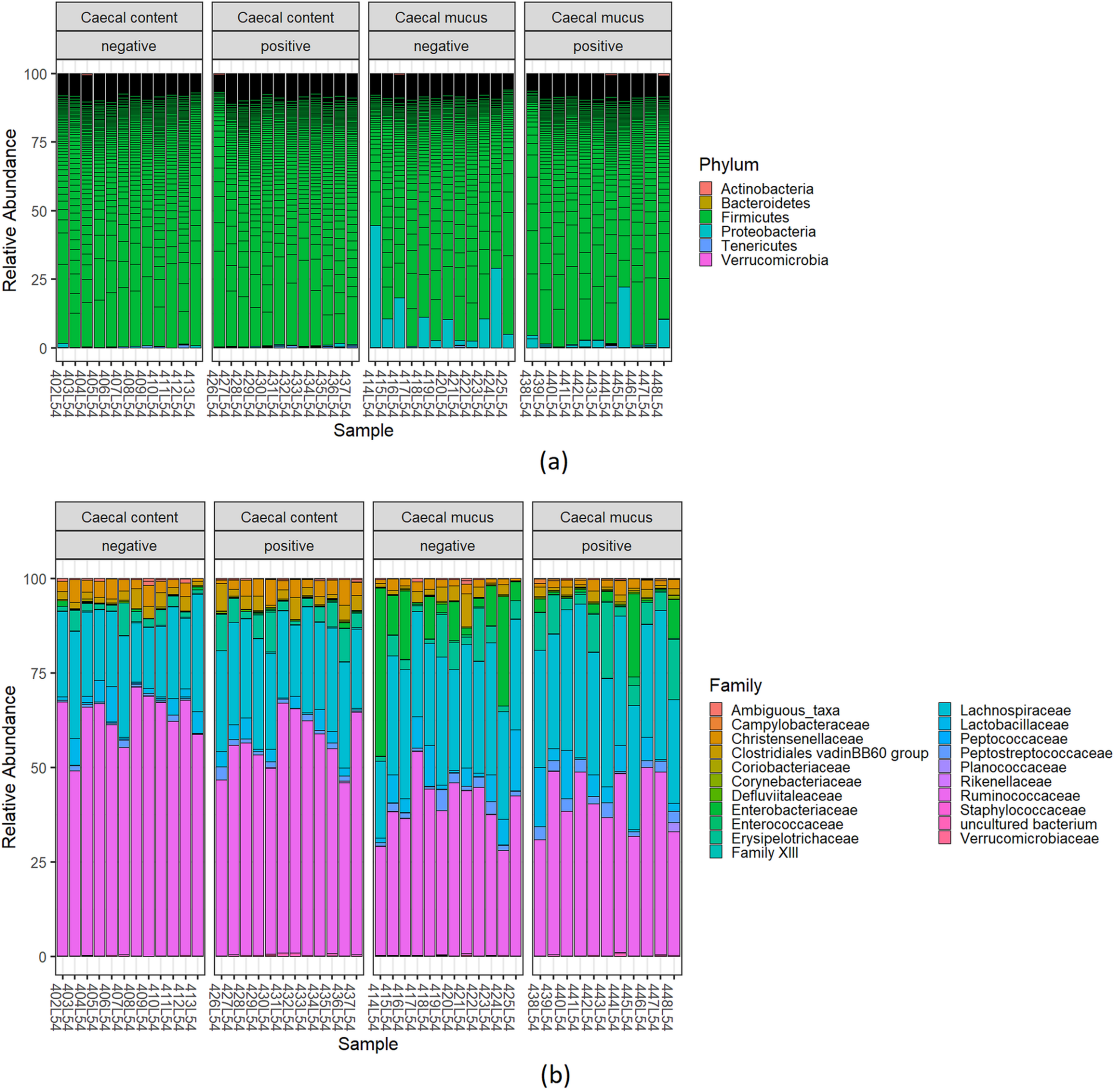
Relative abundance of (a) bacterial phyla and (b) families in luminal and mucosa-associated caecal microbiota of chickens with and without experimental *C. jejuni* infection.

Independent of *C. jejuni* status, *Firmicutes* dominated the microbiota of caecal contents at phylum level, while *Firmicutes* and *Proteobacteria* dominated the microbiota of caecal mucus. Relative abundance of bacterial phyla within all samples is shown in Fig. 2. Luminal and mucosa-associated microbiota showed significant differences regarding bacterial abundance (Table S2).

**Fig. 3.**
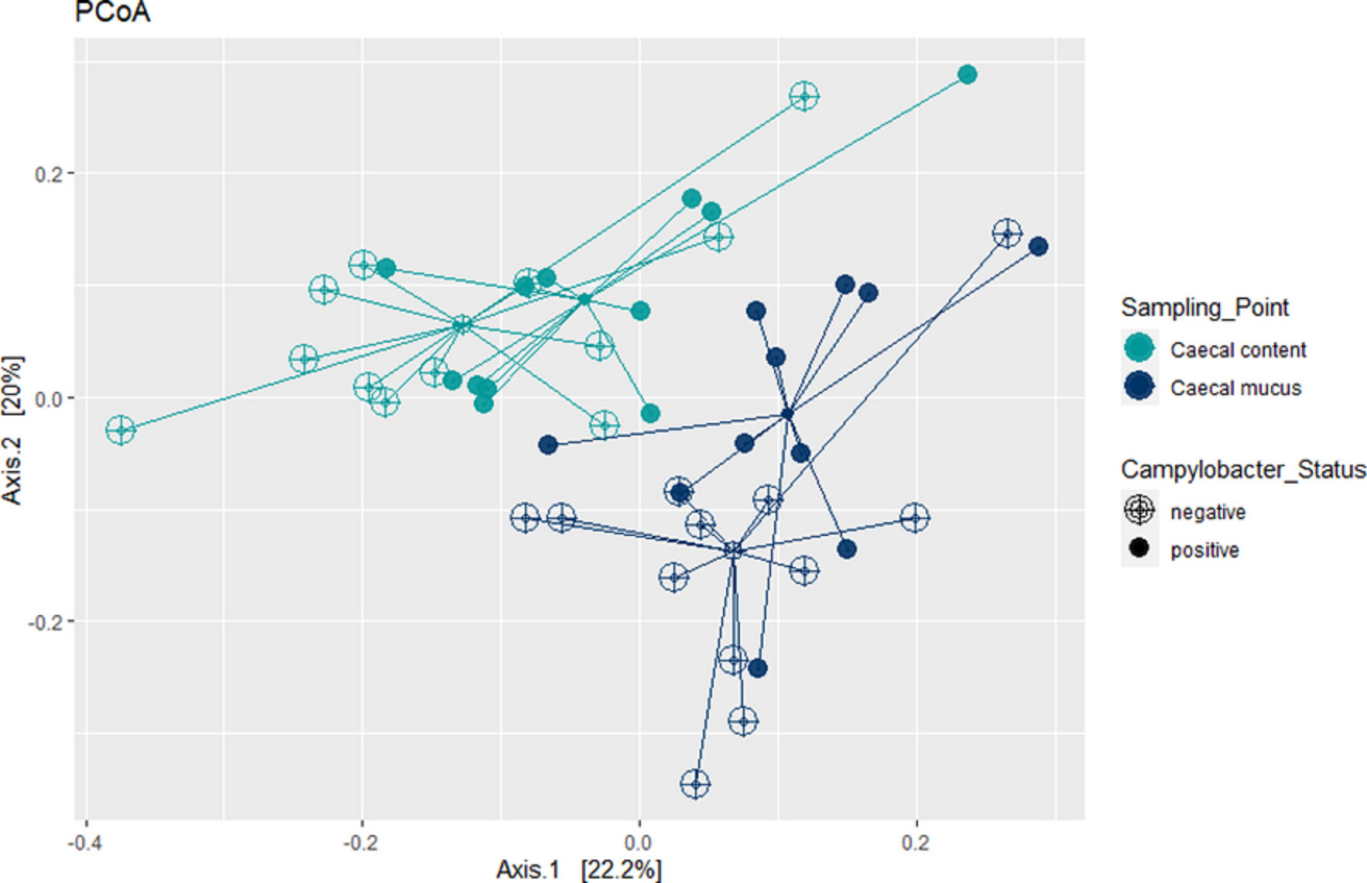
Bray–Curtis dissimilarity-based principal coordinate analysis (PCoA). Each point represents a different bird with *C. jejuni* infection (filled points) or a *C. jejuni *-non-infected bird (open points); coloured lines connect samples of one sampling point: caecal content (green) and caecal mucus (blue).

Both factors, the sampling point (*P*=0.001) and the chickens’ infection status (*P*=0.001) contributed significantly to the differences in microbial composition of the samples. The factor sampling point explained 15.6 % of the sample’s variability, whereas the factor infection status 7.39 % thereof. At both sampling points, luminal and mucosa-associated caecal microbiota, the tested variable *C. jejuni* status altered the microbiota to almost the same extent. The tested variable *C. jejuni* status explained 9.69 % of the microbiota variation in caecal contents (*P*=0.015), while the *C. jejuni* status explained 10.6 % of the microbiota variation in samples of caecal mucus (*P*=0.007). Fig. 3 shows PCoA based on Bray–Curtis dissimilarity of samples separated by the two sampling points, caecal content and mucus, and infection status.

**Fig. 4.**
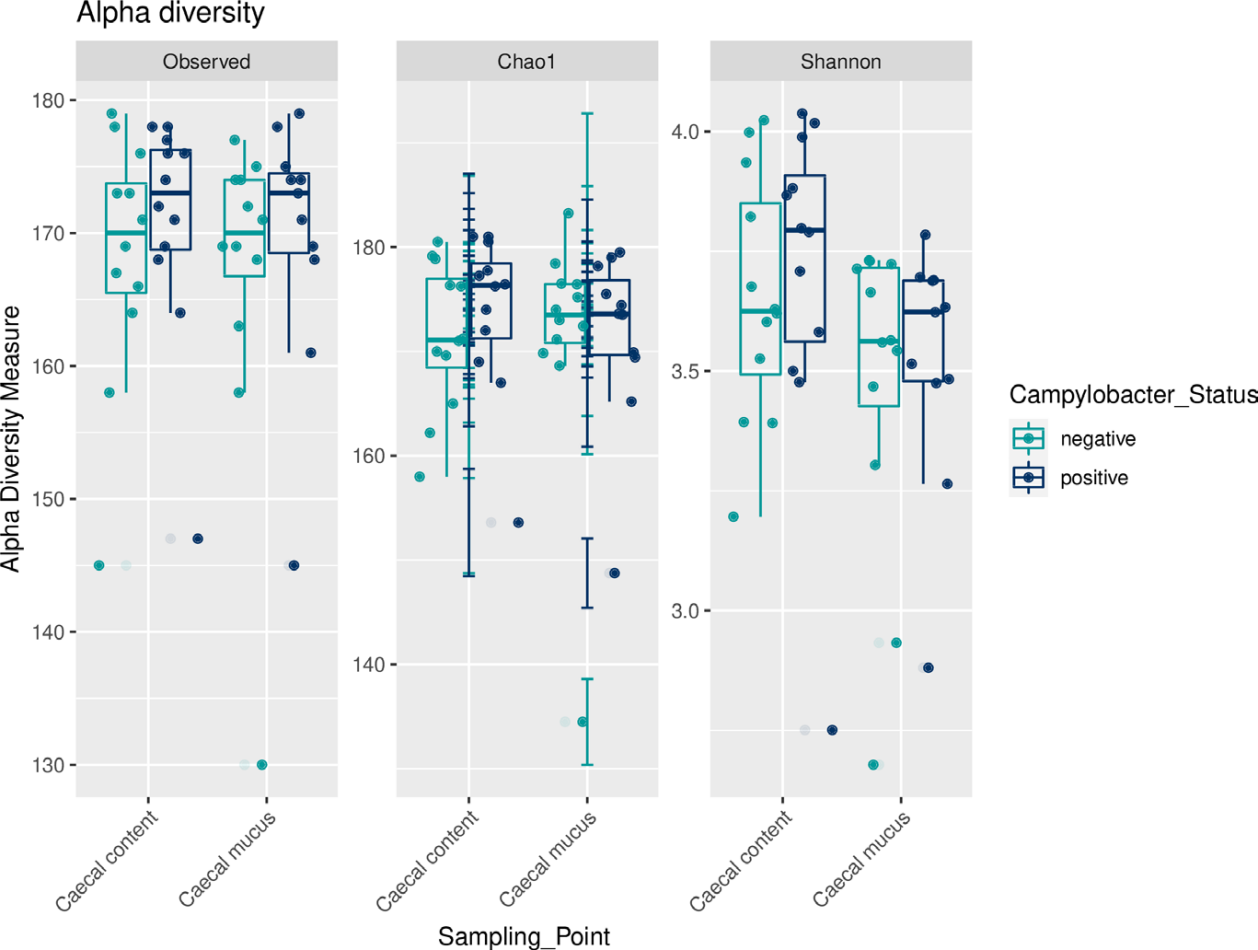
Alpha diversity of luminal and mucosa-associated caecal microbiota of *C. jejuni *-positive and *C. jejuni *-negative chickens. Box-plots showing alpha diversity in samples using the species richness estimators Observed Species, Chao1 and Shannon index.

Pairwise comparisons of measured species richness estimators Observed Species, Chao 1 and Shannon index revealed no statistically significant differences between birds with and without experimental *C. jejuni* infection, neither in luminal nor in mucosa-associated microbiota (Fig. 4).

Multiple testing on normalized counts on each phylum of luminal microbiota yielded no significant differences between animals of different *C. jejuni* status. In contrast to the luminal-associated microbiota, mucosal microbiota showed a shift to an 7.7% points higher relative abundance of the phylum *Firmicutes* in *C. jejuni *-infected birds, as well as an 7.85% points reduction in *Proteobacteria* relative abundance (relative abundance of *Firmicutes* in *C. jejuni *-non-infected birds: 87.3 % and *C. jejuni *-infected birds: 95.0 %; relative abundance of *Proteobacteria* in *C. jejuni *-non-infected birds: 12.1 % and *C. jejuni *-infected birds: 4.25 %; Table S1). Nevertheless, differences in these phyla between non-infected and infected birds were not significant.

**Fig. 5.**
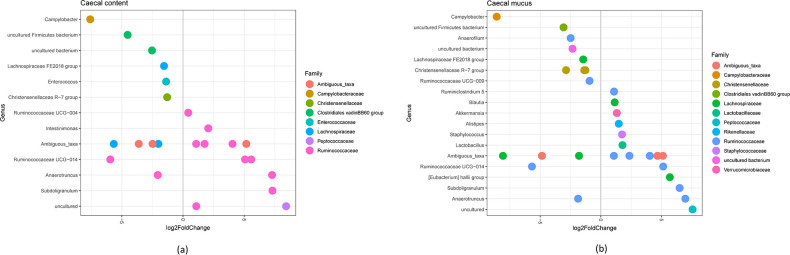
Differential analysis of OTUs using the DESeq2 package (significance threshold for padj <0.05) comparing *C. jejuni *-negative and *C. jejuni *-positive chickens in (a) caecal content, (b) caecal mucus. Each point represents a single OTU grouped by genus and by colour according to which taxonomic family the OTU originates.

At species level, 24 out of 191 OTUs showed a significantly different abundance between caecal contents of *C. jejuni *-infected and non-infected birds (Table S3), while in samples of caecal mucus, 30 OTUs were significantly different between *C. jejuni *-infected and non-infected birds (Table S4). Log_2_-fold changes for these OTUs grouped by genus are shown in Fig. 5.

**Fig. 6.**
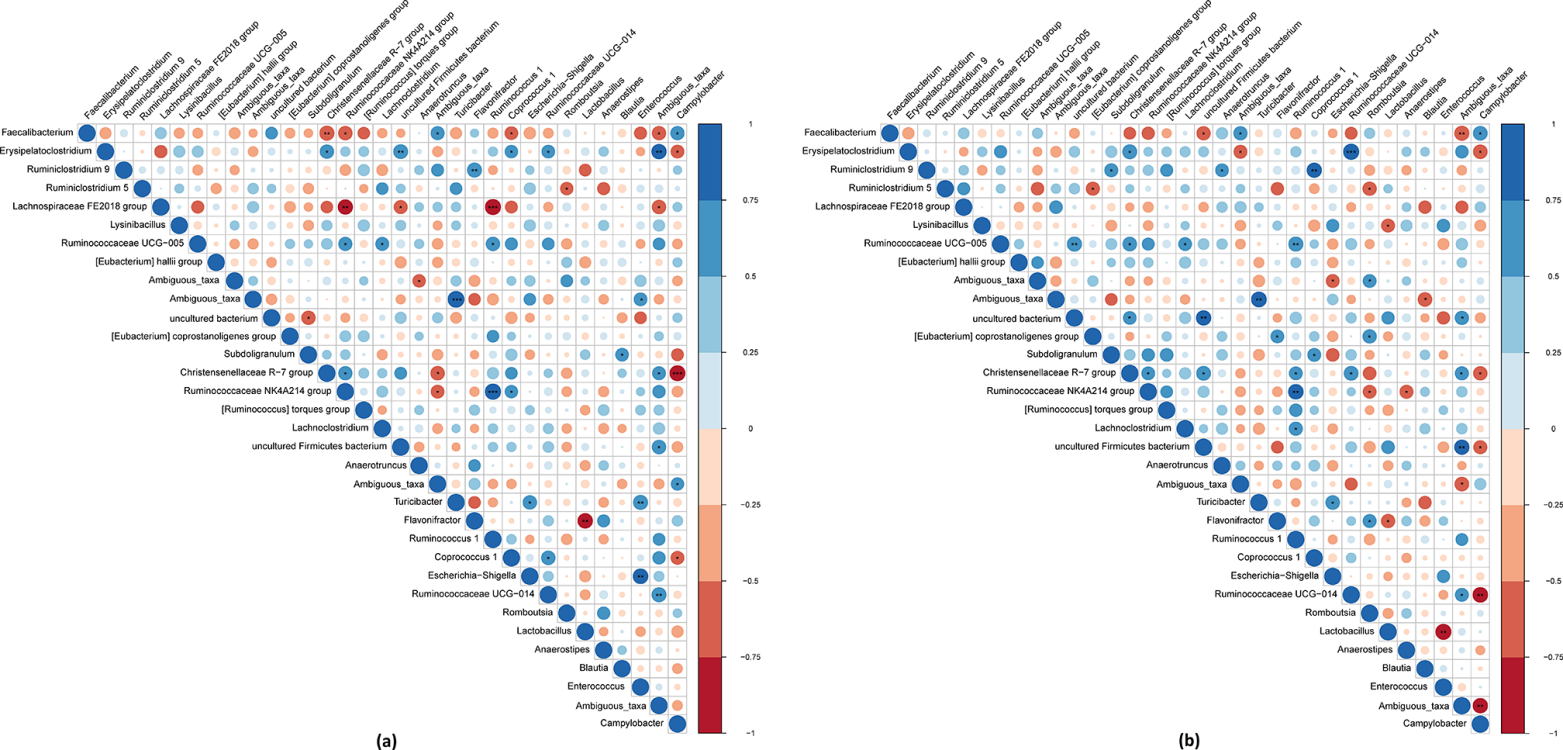
Spearman’s correlation between 33 genera (relative abundance >1 % in at least one sample) of (a) luminal and (b) mucosa-associated microbiota in *C. jejuni *-positive chickens. Positive correlations are displayed in blue and negative correlations in red, while colour intensity and the circle size are proportional to the correlation coefficients. *: *P*-value <0.05, **: *P*-value <0.01, ***: *P*-value <0.001.

With two exceptions, especially sequences of OTUs belonging to genera within the family *Ruminococcaceae* were enriched in caecal contents of non-infected compared to *C. jejuni *-infected chickens (Fig. 5a). These bacterial sequences could be assigned to five different genera, *Ruminococcaceae* UCG-004, *Intestinimonas *, *Ruminococcaceae* UCG-014, *Anaerotruncus* and *Subdoligranulum,* and seem to be depressed as a result of the artificial *Campylobacter* infection. Even if bacterial members of the genus *Ruminococcaceae* UCG-014 were enriched in non-infected birds, one bacterial species of this genus was clearly enriched in caecal contents of *C. jejuni *-infected chickens. Sequences of one OTU assigned to the genus *Enterococcus *, as the only member of this genus in the present study, was enriched *C. jejuni *-infected compared to non-infected chickens. This observation was limited to samples of caecal content. In addition, sequences of OTUs belonging to two genera of the family *Clostridiales* vadinBB60 group were enriched in caecal contents of *C. jejuni *-infected birds. A positive correlation was found between one further unknown genus belonging to this family *Clostridiales* vadinBB60 group and the genus *Campylobacter* in caecal contents of *C. jejuni *-infected chickens (Fig. 6a).

In both, luminal as well as mucosa-associated microbiota, sequences of the bacterial member of an uncultured genus belonging to the family *Peptococcaceae* differed clearly between non-infected and *C. jejuni *-infected chickens (Fig. 5). This genus of the family *Peptococcaceae* is its only bacterial member in the present study*,* whose existence in *C. jejuni *-infected birds was not detected. Not as clear as in caecal contents, but just as noticeable, sequences of OTUs belonging to genera within the family *Ruminococcaceae* were enriched in caecal mucus of non-infected compared to *C. jejuni *-infected chickens (Fig. 5b). Within mucosa-associated microbiota of *C. jejuni *-infected chickens, a strong negative association was found between the genus *Ruminococcaceae* UCG-014 (−0.80, *P*=0.003), one futher unknown genus of the same family (−0.77, *P*=0.005) and the genus *Campylobacter* (Fig. 6b). Additional genera were enriched within mucosa-associated microbiota of non-infected chickens and therefore seem to be depressed due to *Campylobacter* infection only at this site of the caecum; under them *Ruminiclostridium* 5, *Blautia *, *Akkermansia *, *Alistipes *, *Staphylococcus *, *Lactobacillus *, unclassified genus of the order *Clostridiales *, and [Eubacterium] hallii group.

Strengths of an association between bacterial genera and the direction of the relationship with the Spearman’s rank correlation shown in Fig. 6 revealed a strong negative correlation between the genus *Campylobacter* and *Christensenellaceae* R-7 group in both luminal and mucosa- associated caecal microbiota of *C. jejuni *-positive chickens. The strength of association was greater in luminal as in mucosa-associated microbiota (caecal content: −0.84, *P <*0.001; caecal mucus: −0.67, *P=*0.023). Interestingly, this genus was enriched in luminal and mucosa-associated microbiota of *C. jejuni *-infected compared to non-infected chickens (Fig. 5). In addition, there was a positive association of the genera *Campylobacter* and *Faecalibacterium* in luminal as well as in mucosa-associated caecal microbiota of *C. jejuni *-positive chickens (caecal content: 0.66, *P=*0.019; caecal mucus: 0.70, *P=*0.016).

## Discussion

The present infection trial took place under absolutely controlled conditions constituting the prerequisites for an exclusive investigation of possible interactions between caecal microbiota and *Campylobacter *.

### Influence of *C. jejuni* on composition of microbial communities in caeca of chickens

In the present study, it could be shown that the *C. jejuni* infection had significant influence on microbial composition of the samples. Similar results were seen in investigations by Awad *et al*. [27] where the gut microbial communities changed as a result of infection as well. Only 25–36 % of the observed OTUs in the jejunum and caecum were shared between the control and infected birds. Nevertheless, the number of shared OTUs between the control and infected birds at day 21 (1 week after infection) was smaller compared to day 28 (2 weeks after infection). In addition, Thibodeau *et al*. [28] observed that caecal beta-diversity was only moderately modified (visual analysis of the NMDS graph) albeit significantly after *C. jejuni* colonisation (UniFrac significance *P*=0.001). This shows that the impact of *Campylobacter* on microbiota composition seems to vary according to the age of the chickens and the time passed after infection, being less prominent for older chickens and the time after infection.

Alpha diversity did not differ between birds with and without experimental *C. jejuni* infection in the present study, neither in luminal nor in mucosa-associated microbiota. Still, *C. jejuni *-infected birds showed slightly higher richness and evenness. These results are consistent with previous investigations. Thibodeau *et al*. [28] observed no impact of *C. jejuni* colonization on alpha diversity in caecal microbiota of Ross 308 chickens (means of Chao1 index of *C. jejuni *-negative birds: 644 and *C. jejuni *-positive birds: 663; means of Shannon index of *C. jejuni *-negative birds: 3.8 and *C. jejuni *-positive birds: 3.7). In contrast, Awad *et al*. [27] noticed a significantly higher species richness in caecal contents of infected Ross 308 chickens 14 days after infection [Sobs, Chao1, and ACE (*P*=0.047)]. The authors concluded from the observed higher diversity, an indication that the *Campylobacter* infection increased the microbiota complexity. One further study investigated the microbiota of chickens at an industrial farm environment with the aim to investigate *Campylobacter* appearance within a natural habitat setting [29]. McKenna *et al*. [29] also recognized that the presence of *Campylobacter* was linked with an increased microbial diversity (*P* <0.05). At this point, however, the question arises whether with the introduction of *Campylobacter* onto the farm, other bacteria were introduced as well, that may have brought about this observation on microbiota diversity. In the present study, the risk of bacterial introduction from outside the infection unit was reduced to a minimum, which is why the results of the present study can be seen under the sole factor of a *Campylobacter* infection.

Another difference between the studies could reveal an additional factor that should be taken into account in studies on *Campylobacter* infections. In the present study and the study conducted by Thibodeau *et al*. [28], 25 and 21 days passed between experimental *Campylobacter* infection and intestinal microbiota analyses, respectively, in contrast to Awad *et al*. [27], who investigated the microbiota already 14 days after infection. Additionally, Awad *et al*. [27] investigated the intestinal microbiota at a younger chicken age (28 days) compared to the present study (42 days) and Thibodeau *et al*. [28], who collected the samples at an age of 35 days. Taking these observations and the results of the present study into account, it seems that age at the time of examination and the time passed after *Campylobacter* infection are two important factors that should be considered when investigating intestinal microbiota and its impact on *Campylobacter* and vice versa. Similar to the comments on microbiota composition, the impact of *Campylobacter* on microbiota diversity might vary according to the age of the chickens and the time passed after infection, being less prominent for older chickens and the time after infection.

Mucosal microbiota of *C. jejuni *-infected birds in the present study showed a higher relative abundance of the phylum *Firmicutes* and a lower relative abundance of *Proteobacteria *. Even if the differences in these phyla between non-infected and infected birds were not significant, similar observations were previously made in other studies for luminal-associated microbiota of the caecum. Awad *et al*. [27] observed similar shifts in the two major phyla in caecal contents towards an enrichment of *Firmicutes* (relative abundance in control birds: 70.86 % and infected birds: 97.65 %; *P*=0.031) with a concomitant reduction in *Proteobacteria* (relative abundance in control birds: 4.42 % and infected birds: 0.71 %; *P*=0.029) due to *Campylobacter* colonization at day 28 of life. Besides these major shifts, Awad *et al*. [27] also observed an alteration in low abundant phyla (e.g. *Actinobacteria* and *Tenericutes *) by *Campylobacter* infection. The authors assume that these shifts could also disequilibrate the microbiome composition. In the current study, significant differences due to *C. jejuni* infection were only seen in caecal mucus for the low abundant phyla *Bacteroidetes* and *Verrucomicrobia *.

### Enrichment of bacteria within caecal microbiota of chickens due to *Campylobacter* infection

The genus *Enterococcus* seemed to be enriched due to *C. jejuni* infection. In investigations by Pandit *et al*. [30], a repeated positive correlation was found between *Campylobacter* and *Enterococcus *, well in line with the present study. In Kaakoush *et al*. [31] *Enterococcus* was associated with the presence of *C. jejuni* in the chicken gastrointestinal tract as well. However, in contrast to the present study, also a decreased relative abundance of *Enterococcus* with *C. jejuni* presence in caecal contents was observed [27, 31, 32]. Within the order *Lactobacillales* and the family *Enterococcaceae *, the genus *Enterococcus* is known for its fermentative metabolism with the predominant end product of glucose: l-lactate [33]. A variety of bacterial species have been used as probiotics in poultry including *Enterococcus* [34]. On the other hand, infections with pathogen *Enterococcus cecorum* strains form an important emerging disease in modern broiler chicken lines associated with arthritis and osteomyelitis, leading to high mortality rates [35–37]. There are indications that the presence of certain bacterial species of the microbiota may encourage the presence of *Enterococci* by providing nutrients. Vancomycin-resistant *Enterococci* are unable to ferment mucin-derived complex polysaccharides; their growth was supported after a pre-digestion of mucins with enzyme mixtures obtained from human faeces, which resulted in a release of monosaccharides [38]. Some intestinal mucolytic bacteria use their specific enzymatic activities to degrade mucins and release monosaccharides attached to the mucin glycoproteins that can be used by other resident bacteria [39]. It has been shown that *C. jejuni* upregulates putative mucin-degrading enzymes in the presence of mucins [40]. For this reason, it can be suspected that in consequence of the presence of *C. jejuni,* mucins were degraded and monosaccharides released, which may have been used by *Enterococcus *. In any case, what can be assumed from the results of the present study is that environmental changes due to the presence of *C. jejuni* promoted the growth of *Enterococcus.*

In addition, the *Clostridiales* vadin BB60 group seem to benefit from a *Campylobacter* infection as its abundance was enriched in caecal contents of *C. jejuni *-infected compared to non-infected birds of the present study. This genus is known to be a later colonizer of the chickens caecum, however this genus is poorly classified and little is known about its metabolism or role within microbiota [41].

A positive association between *Faecalibacterium* and *Campylobacter,* as seen in the present study, was observed before in other studies with *C. jejuni *-infected chickens [28, 31]. The abundance of *Faecalibacterium* increased within the caecum of chickens that were colonized by *C. jejuni* [28]*. Faecalibacterium* reported in chickens, its 16S rRNA sequence is only 96–97% similar to 16S rRNA of *Faecalibacterium prausnitzii* from humans, however, both human and chicken *Faecalibacterium* isolates are efficient butyrate producers [42]. This ability of *Faecalibacterium* to produce butyrate appears to be in contradiction with its positive association with *C. jejuni* [28] as it was reported to be detrimental to *C. jejuni* [43, 44]. The mutualistic relationship between these two bacterial genera (* Faecalibacterium* and *Campylobacter *), seems to occur by means of an intermediate microbial conglomerate (* Limnobacter *, *Parabacteroides *, *Pseudomonadaceae *, *Sutterella *, *Sphingobium* and *Oxalobacteraceae *) that appears to modulate their mutual interactions [45]. The authors discuss that their identified network topology suggests the possibility of a commensalistic relationship between *Faecalibacterium* and this intermediate microbial conglomerate; conversely, an amensalistic relationship could be in place between the intermediate microbial conglomerate and *Campylobacter* [45]. Nevertheless, the reverse relation between both genera was found as well [46].

### Negative association between bacteria and the genus *Campylobacter* or in the context of the *Campylobacter* infection

The existence of members belonging to the family *Peptococcaceae* could not be demonstrated in *C. jejuni *-infected birds in the present study. *Peptococcaceae* colonize the caecum of chickens in the third week of life [41, 47] and can be found in caecal digesta as well as mucosa samples [48], being more abundant in the lumen [10]. *Peptococcaceae* found in human and animal intestines are obligate anaerobes with a fermentative metabolism, producing caproic acid as a terminal metabolite of glucose [49]. To our knowledge, a connection between the presence or absence of the family *Peptococcaceae* and an infection with *C. jejuni* has not yet been described.

Various genera of the family *Ruminococcaceae* seemed to be depressed under *C. jejuni* infection in the present study. Additionally, a strong negative relation was found between two genera of this family and the genus *Campylobacter *. Other studies also suggest that the co-occurrence in high levels of certain genera of this family and *C. jejuni* does not seem to be compatible. Only 16 weeks after oral infection with *C. jejuni *, the *Ruminococcaceae* family exhibited an increased relative abundance that plateaued and thus was stably present within the caecal microbiota [50]. This occurred at the same time when most laying hens became negative and remained negative for *C. jejuni* [50]. The role of bacterial members of the family *Ruminococcaceae* within the intestinal microbiota of chickens was reviewed by Rychlik [42]. *Ruminococcaceae* represent major butyrate producers of which the vegetative cells are highly sensitive to oxygen, and are therefore among the first bacteria to disappear from gut microbiota during inflammatory diseases due to the production of reactive oxygen species by macrophages and granulocytes [42]. This means that in most cases, the decrease of *Ruminococcaceae* is not the cause of the inflammation but its consequence [42].

Furthermore, a strong negative correlation between the genus *Campylobacter* and *Christensenellaceae* R-7 group of the family *Christensenellaceae* was seen in the present study. The name *Christensenellaceae* is derived from the isolate named *Christensenella minuta *, a saccharolytic bacterium with acetic acid and a small amount of butyric acid as end products of glucose fermentation [51]. *Christensenellaceae* form a family of bacteria within the phylum *Firmicutes *, class *Clostridia *, order *Clostridiales* [51]. In 2015, Thibodeau *et al*. [28] observed a relationship between an unclassified genus of the family *Christensenellaceae* and *C. jejuni* colonization of chickens. This unknown genus as well as the family *Christensenellaceae* decreased in relative abundance in birds colonized by *C. jejuni *, but its involvement in chicken health was still unknown [28]. In the meantime, *Christensenellaceae* have emerged as an important player in human health [52], and more knowledge has been gained about this family within the microbiota of chickens as well. *Christensenellaceae* are later colonizers of the caecal microbiota, being most apparent from day 14 post-hatch [41], while the chicken’s breed has been shown to have an impact on its abundance [41, 53, 54]. Even if Richards *et al*. [41] observed a higher abundance of *Christensenellaceae* in the chicken’s mucus, the present study revealed a higher relative abundance of *Christensenellaceae* in luminal compared to mucosa-associated microbiota, this family being the fourth most common one in caecal contents of chickens (after *Ruminococcaceae *, *Lachnospiraceae* and *Erysipelotrichaceae *). Recently, an association between *Christensenellaceae* R-7 group and another enteric pathogen was found in chickens [55]. The family *Christensenellaceae* and its genus *Christensenellaceae* R-7 group was more abundant in low compared to high *Salmonella enterica* Enteritidis-carrying chickens and found to be higher in a White Leghorn inbred line, which are more resistant to *Salmonella* [55]. In contrast to Thibodeau *et al*. [28], we observed an enrichment of bacterial sequences of the genus *Christensenellaceae* R-7 group in *C. jejuni *-infected compared to non-infected birds. Even if the differences were significant, the log_2_-fold changes were small (with one exception <2). In addition, the relative abundance of the genus *Christensenellaceae* R-7 group was significantly negatively related to the relative abundance of *Campylobacter* in *C. jejuni *-infected chickens in luminal and mucosa-associated microbiota. It can be hypothesized that members of both families compete for substrate. Amino acids are used by *C. jejuni* as carbon and energy sources (reviewed in [56]) or directly for protein synthesis [57], while *Christensenellaceae* have recently been positively associated with gut metabolites typical of amino acid degradation [58].

## Conclusion

In the present study, exclusive interactions between caecal luminal and mucosa-associated microbiota and *C. jejuni* could be assessed in an infection model with high explanatory power that was achieved by the applied biosecurity measures and assurance of identical conditions between both groups that were only separated by a continuous wall. At the same time, chickens were obtained from a commercial hatchery, feeding was adapted to usual field conditions, common bedding material was used and different *C. jejuni* prevalences were generated via the boxes in order to simulate practical conditions. Based on the obtained data, it can be concluded that *C. jejuni* infection contributed significantly to the differences in microbial composition, while the contribution to mucosa-associated bacteria was greater compared to its influence on luminal microbiota. The negative correlation found between the genus *Christensenellaceae* R-7 group and *Campylobacter* in *C. jejuni *-colonized chickens makes this family highly interesting for further studies that aim to find control options for *Campylobacter* infections in chickens.

## Supplementary Data

Supplementary material 1
