# Supplementary material for: Luminal and mucosa-associated caecal microbiota of chickens after experimental Campylobacter jejuni infection in the absence of Campylobacter-specific phages of group II and III
Source: Microb Genom. 2022 Oct 3;8(10):mgen000874. doi: 10.1099/mgen.0.000874 (PMC9676049; doi:10.1099/mgen.0.000874)
Supplement: Supplementary material 1 [file mgen-8-874-s001.pdf]

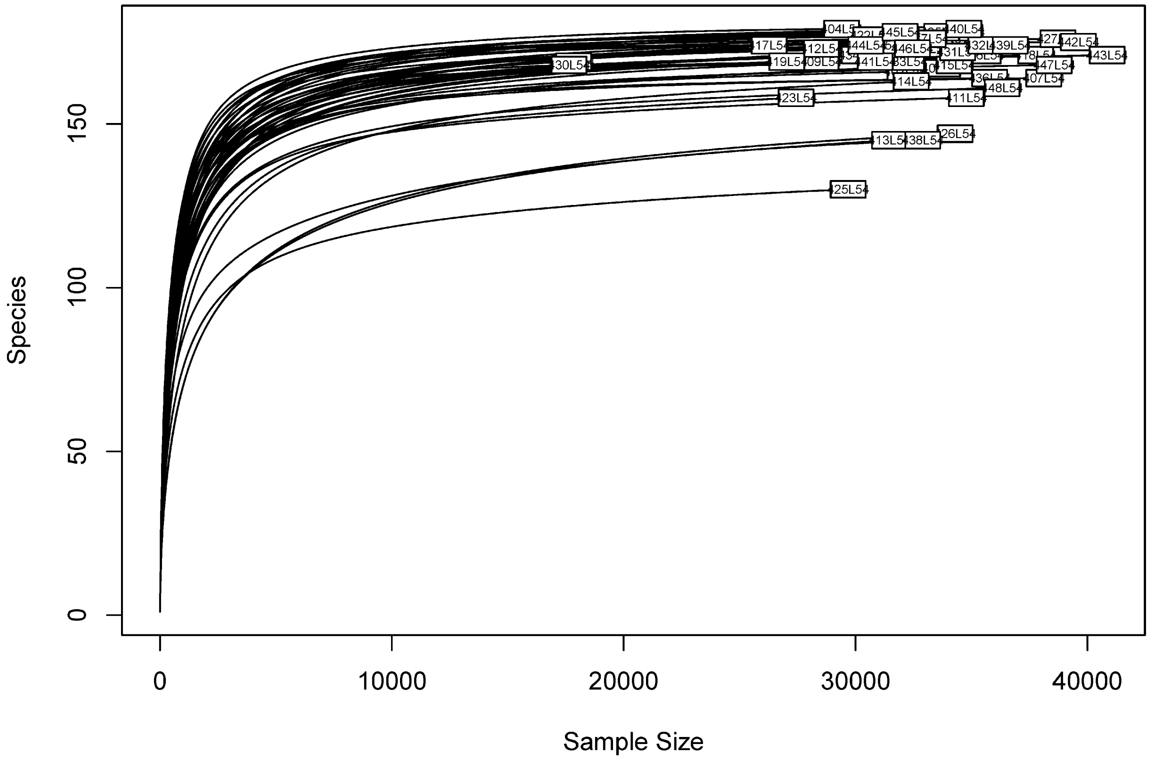

Figure S1. Rarefraction curves for the 47 samples.

Table S1. Relative abundance (%) of bacterial phyla in luminal or mucosa-associated microbiota depending on *C. jejuni* status.

**Caecal content**			
Phylum	*C. jejuni*-non-inf.	*C. jejuni*-inf.	BH-adjusted *p*-value
Actinobacteria	0.240	0.293	n.s.
Bacteroidetes	0.021	0.039	n.s.
Firmicutes	99.1	98.7	n.s.
Proteobacteria	0.265	0.345	n.s.
Tenericutes	0.363	0.524	n.s.
Verrucomicrobia	0.033	0.059	n.s.
**Caecal mucus**			
Phylum	*C. jejuni*-non-inf.	*C. jejuni*-inf.	BH-adjusted *p*-value
Actinobacteria	0.253	0.409	n.s.
Bacteroidetes	0.056	0.022	0.0183
Firmicutes	87.3	95.0	n.s.
Proteobacteria	12.1	4.25	n.s.
Tenericutes	0.151	0.303	n.s.
Verrucomicrobia	0.085	0.037	0.0183

n.s.: not significant; *p*-values were adjusted by the Benjamini and Hochberg (BH) method to control for the false discovery rate (FDR) of 5%.

Table S2. Comparison of bacterial relative abundance in samples of luminal and mucosa-associated microbiota (at taxonomic level, family).

Phylum	Class	Order	Family	BH-adjusted *p*-value
Firmicutes	Clostridia	Clostridiales	Ruminococcaceae	**0.0004**
Firmicutes	Clostridia	Clostridiales	Family XIII	**0.0004**
Firmicutes	Clostridia	Clostridiales	Lachnospiraceae	**0.0004**
Firmicutes	Clostridia	Clostridiales	Christensenellaceae	**0.0004**
Firmicutes	Bacilli	Bacillales	Staphylococcaceae	**0.0004**
Proteobacteria	Gammaproteobacteria	Enterobacteriales	Enterobacteriaceae	**0.0004**
Firmicutes	Clostridia	Clostridiales	Peptostreptococcaceae	**0.001**
Firmicutes	Bacilli	Lactobacillales	Enterococcaceae	**0.003**
Firmicutes	Bacilli	Lactobacillales	Lactobacillaceae	0.0675
Firmicutes	Erysipelotrichia	Erysipelotrichales	Erysipelotrichaceae	0.1423
Tenericutes	Mollicutes	Mollicutes RF9	uncultured bacterium	0.1618
Actinobacteria	Actinobacteria	Corynebacteriales	Corynebacteriaceae	0.1774
Tenericutes	Mollicutes	Mollicutes RF9	Ambiguous_taxa	0.1774
Firmicutes	Clostridia	Clostridiales	Clostridiales vadinBB60 group	0.1774
Proteobacteria	Epsilonproteobacteria	Campylobacterales	Campylobacteraceae	0.1811
Firmicutes	Clostridia	Clostridiales	Peptococcaceae	0.2258
Verrucomicrobia	Verrucomicrobiae	Verrucomicrobiales	Verrucomicrobiaceae	0.4053
Firmicutes	Clostridia	Clostridiales	Defluviitaleaceae	0.4546
Firmicutes	Clostridia	Clostridiales	Ambiguous_taxa	0.4546
Bacteroidetes	Bacteroidia	Bacteroidales	Rikenellaceae	0.5059
Firmicutes	Bacilli	Bacillales	Planococcaceae	0.5726
Actinobacteria	Coriobacteriia	Coriobacteriales	Coriobacteriaceae	0.9491

Table S3. OTUs with significant (padj < 0.05) different abundance between chickens of different *C. jejuni* status in samples of caecal content.

**Caecal content**								
OTU_ID	log2FoldChange	*p*-value	padj	Phylum	Class	Order	Family	Genus
OTU_107	8.42	1.30E-11	2.33E-09	Firmicutes	Clostridia	Clostridiales	Peptococcaceae	uncultured
OTU_90	-7.58	2.45E-11	2.33E-09	Proteobacteria	Epsilonproteobacteria	Campylobacterales	Campylobacteraceae	Campylobacter
OTU_80	7.28	1.22E-10	7.73E-09	Firmicutes	Clostridia	Clostridiales	Ruminococcaceae	Anaerotruncus
OTU_8	4.04	5.62E-09	2.32E-07	Firmicutes	Clostridia	Clostridiales	Ruminococcaceae	Ambiguous_taxa
OTU_137	-5.66	6.10E-09	2.32E-07	Firmicutes	Clostridia	Clostridiales	Lachnospiraceae	Ambiguous_taxa
OTU_119	-5.94	1.68E-08	5.33E-07	Firmicutes	Clostridia	Clostridiales	Ruminococcaceae	Ruminococcaceae UCG-014
OTU_145	7.30	7.71E-08	2.09E-06	Firmicutes	Clostridia	Clostridiales	Ruminococcaceae	Subdoligranulum
OTU_66	-2.01	1.45E-07	3.43E-06	Firmicutes	Clostridia	Clostridiales	Lachnospiraceae	Ambiguous_taxa
OTU_515	-4.53	4.55E-07	9.61E-06	Firmicutes	Clostridia	Clostridiales	Clostridiales vadinBB60 group	uncultured Firmicutes bacterium
OTU_129	5.17	2.56E-05	0.0005	Firmicutes	Clostridia	Clostridiales	Ambiguous_taxa	Ambiguous_taxa
OTU_227	5.56	3.27E-05	0.0006	Firmicutes	Clostridia	Clostridiales	Ruminococcaceae	Ruminococcaceae UCG-014
OTU_540	2.06	6.01E-05	0.001	Firmicutes	Clostridia	Clostridiales	Ruminococcaceae	Intestinimonas
OTU_367	-2.07	0.0002	0.002	Firmicutes	Clostridia	Clostridiales	Ruminococcaceae	Anaerotruncus
OTU_196	-2.53	0.0004	0.006	Firmicutes	Clostridia	Clostridiales	Clostridiales vadinBB60 group	uncultured bacterium
OTU_151	-2.49	0.0012	0.0147	Tenericutes	Mollicutes	Mollicutes RF9	Ambiguous_taxa	Ambiguous_taxa
OTU_72	-1.38	0.0012	0.0148	Firmicutes	Bacilli	Lactobacillales	Enterococcaceae	Enterococcus
OTU_71	5.07	0.0020	0.0206	Firmicutes	Clostridia	Clostridiales	Ruminococcaceae	Ruminococcaceae UCG-014
OTU_189	1.76	0.0018	0.0206	Firmicutes	Clostridia	Clostridiales	Ruminococcaceae	Ambiguous_taxa
OTU_44	1.08	0.0025	0.0246	Firmicutes	Clostridia	Clostridiales	Ruminococcaceae	uncultured
OTU_388	0.43	0.0028	0.0268	Firmicutes	Clostridia	Clostridiales	Ruminococcaceae	Ruminococcaceae UCG-004
OTU_178	-3.62	0.0035	0.0320	Firmicutes	Clostridia	Clostridiales	Ambiguous_taxa	Ambiguous_taxa
OTU_41	1.07	0.0043	0.0374	Firmicutes	Clostridia	Clostridiales	Ruminococcaceae	Ambiguous_taxa
OTU_122	-1.56	0.0052	0.0427	Firmicutes	Clostridia	Clostridiales	Lachnospiraceae	Lachnospiraceae FE2018 group
OTU_121	-1.30	0.0063	0.0496	Firmicutes	Clostridia	Clostridiales	Christensenellaceae	Christensenellaceae R-7 group

Table S4. OTUs with significant (padj < 0.05) different abundance between chickens of different *C. jejuni* status in samples of caecal mucus.

OTU_ID	log2FoldChange	*p*-value	padj	Phylum	Class	Order	Family	Genus
OTU_90	-8.59	3.07E-19	5.87E-17	Proteobacteria	Epsilonproteobacteria	Campylobacterales	Campylobacteraceae	Campylobacter
OTU_137	-8.07	7.10E-17	6.78E-15	Firmicutes	Clostridia	Clostridiales	Lachnospiraceae	Ambiguous_taxa
OTU_80	6.96	1.43E-10	9.12E-09	Firmicutes	Clostridia	Clostridiales	Ruminococcaceae	Anaerotruncus
OTU_107	7.57	4.48E-10	2.14E-08	Firmicutes	Clostridia	Clostridiales	Peptococcaceae	uncultured
OTU_8	4.04	4.54E-08	1.73E-06	Firmicutes	Clostridia	Clostridiales	Ruminococcaceae	Ambiguous_taxa
OTU_66	-1.80	2.74E-07	8.73E-06	Firmicutes	Clostridia	Clostridiales	Lachnospiraceae	Ambiguous_taxa
OTU_145	6.50	4.52E-07	1.23Ee-05	Firmicutes	Clostridia	Clostridiales	Ruminococcaceae	Subdoligranulum
OTU_119	-5.68	6.73E-07	1.61E-05	Firmicutes	Clostridia	Clostridiales	Ruminococcaceae	Ruminococcaceae UCG-014
OTU_112	1.78	1.72E-06	3.65E-05	Firmicutes	Bacilli	Lactobacillales	Lactobacillaceae	Lactobacillus
OTU_166	4.67	7.51E-05	0.0014	Firmicutes	Clostridia	Clostridiales	Ambiguous_taxa	Ambiguous_taxa
OTU_178	-4.86	0.0001	0.0024	Firmicutes	Clostridia	Clostridiales	Ambiguous_taxa	Ambiguous_taxa
OTU_129	5.12	0.0002	0.0027	Firmicutes	Clostridia	Clostridiales	Ambiguous_taxa	Ambiguous_taxa
OTU_227	5.14	0.0002	0.0027	Firmicutes	Clostridia	Clostridiales	Ruminococcaceae	Ruminococcaceae UCG-014
OTU_51	1.09	0.0002	0.0031	Firmicutes	Clostridia	Clostridiales	Ruminococcaceae	Ruminiclostridium 5
OTU_139	1.32	0.0003	0.0035	Verrucomicrobia	Verrucomicrobiae	Verrucomicrobiales	Verrucomicrobiaceae	Akkermansia
OTU_69	-0.93	0.0004	0.0043	Firmicutes	Clostridia	Clostridiales	Ruminococcaceae	Ruminococcaceae UCG-009
OTU_179	1.48	0.001	0.0075	Bacteroidetes	Bacteroidia	Bacteroidales	Rikenellaceae	Alistipes
OTU_140	1.75	0.001	0.0075	Firmicutes	Bacilli	Bacillales	Staphylococcaceae	Staphylococcus
OTU_565	-2.49	0.001	0.0075	Firmicutes	Clostridia	Clostridiales	Ruminococcaceae	Anaerofilum
OTU_515	-3.07	0.001	0.0092	Firmicutes	Clostridia	Clostridiales	Clostridiales vadinBB60 group	uncultured Firmicutes bacterium
OTU_367	-1.88	0.001	0.0094	Firmicutes	Clostridia	Clostridiales	Ruminococcaceae	Anaerotruncus
OTU_41	1.07	0.0013	0.0116	Firmicutes	Clostridia	Clostridiales	Ruminococcaceae	Ambiguous_taxa
OTU_70	-1.37	0.0017	0.0140	Firmicutes	Clostridia	Clostridiales	Christensenellaceae	Christensenellaceae R-7 group
OTU_563	5.69	0.0021	0.0169	Firmicutes	Clostridia	Clostridiales	Lachnospiraceae	[Eubacterium] hallii group
OTU_493	2.35	0.0033	0.0250	Firmicutes	Clostridia	Clostridiales	Ruminococcaceae	Ambiguous_taxa
OTU_146	-2.33	0.0035	0.0257	Tenericutes	Mollicutes	Mollicutes RF9	uncultured bacterium	uncultured bacterium
OTU_122	-1.46	0.0038	0.0262	Firmicutes	Clostridia	Clostridiales	Lachnospiraceae	Lachnospiraceae FE2018 group
OTU_77	-2.87	0.0038	0.0262	Firmicutes	Clostridia	Clostridiales	Christensenellaceae	Christensenellaceae R-7 group
OTU_29	1.14	0.0042	0.0279	Firmicutes	Clostridia	Clostridiales	Lachnospiraceae	Blautia
OTU_121	-1.27	0.0071	0.0453	Firmicutes	Clostridia	Clostridiales	Christensenellaceae	Christensenellaceae R-7 group

Table S5. Real-time PCR results

Sample No.	ct value		Campylobacter detection
	CPG III+CPGII/III-mulitplex	CPGII	
positive control serial dilution	CPGIII: 16.89-35; CPGII/III 16.58-35	16.2-35	
377	-	-	positive
381	-	-	positive
383	-	-	positive
384	-	-	positive
386	-	-	positive
387	-	-	positive
393	-	-	positive
394	-	-	positive
395	-	-	positive
397	-	-	positive
399	-	-	positive
454	-	-	positive
379	-	35; no slope	negative
380	-	32; no slope	negative
385	-	32; no slope	negative
388	-	28; no slope	negative
389	-	32; no slope	negative
390	-	33; no slope	negative
391	-	-	negative
392	-	33; no slope	negative
396	-	35; no slope	negative
400	-	-	negative
459	-	35; no slope	negative
462	-	-	negative

Table S6. Sample overview

#SampleID	Description	Campylobacter_Status	Sampling_Point
402L54	TiHoJH_1	negative	Caecal content
403L54	TiHoJH_2	negative	Caecal content
404L54	TiHoJH_3	negative	Caecal content
405L54	TiHoJH_4	negative	Caecal content
406L54	TiHoJH_5	negative	Caecal content
407L54	TiHoJH_6	negative	Caecal content
408L54	TiHoJH_7	negative	Caecal content
409L54	TiHoJH_8	negative	Caecal content
410L54	TiHoJH_9	negative	Caecal content
411L54	TiHoJH_10	negative	Caecal content
412L54	TiHoJH_11	negative	Caecal content
413L54	TiHoJH_12	negative	Caecal content
414L54	TiHoJH_13	negative	Caecal mucus
415L54	TiHoJH_14	negative	Caecal mucus
416L54	TiHoJH_15	negative	Caecal mucus
417L54	TiHoJH_16	negative	Caecal mucus
418L54	TiHoJH_17	negative	Caecal mucus
419L54	TiHoJH_18	negative	Caecal mucus
420L54	TiHoJH_19	negative	Caecal mucus
421L54	TiHoJH_20	negative	Caecal mucus
422L54	TiHoJH_21	negative	Caecal mucus
423L54	TiHoJH_22	negative	Caecal mucus
424L54	TiHoJH_23	negative	Caecal mucus
425L54	TiHoJH_24	negative	Caecal mucus
426L54	TiHoJH_25	positive	Caecal content
427L54	TiHoJH_26	positive	Caecal content
428L54	TiHoJH_27	positive	Caecal content
429L54	TiHoJH_28	positive	Caecal content
430L54	TiHoJH_29	positive	Caecal content
431L54	TiHoJH_30	positive	Caecal content
432L54	TiHoJH_31	positive	Caecal content
433L54	TiHoJH_32	positive	Caecal content
434L54	TiHoJH_33	positive	Caecal content
435L54	TiHoJH_34	positive	Caecal content
436L54	TiHoJH_35	positive	Caecal content
437L54	TiHoJH_36	positive	Caecal content
438L54	TiHoJH_37	positive	Caecal mucus
439L54	TiHoJH_38	positive	Caecal mucus
440L54	TiHoJH_39	positive	Caecal mucus
441L54	TiHoJH_40	positive	Caecal mucus
442L54	TiHoJH_41	positive	Caecal mucus
443L54	TiHoJH_42	positive	Caecal mucus
444L54	TiHoJH_43	positive	Caecal mucus
445L54	TiHoJH_44	positive	Caecal mucus
446L54	TiHoJH_45	positive	Caecal mucus
447L54	TiHoJH_46	positive	Caecal mucus
448L54	TiHoJH_47	positive	Caecal mucus
449L54	TiHoJH_48	positive	Caecal mucus
